# Context-Aware Retrieval-Augmented Generation for Artificial Intelligence in Urology

**DOI:** 10.7759/cureus.88167

**Published:** 2025-07-17

**Authors:** Aadhitya Sriram, Maheswaran N, Bose Sundan, Sriram Krishnamoorthy

**Affiliations:** 1 Department of Computer Science and Engineering, College of Engineering, Guindy, Anna University, Chennai, IND; 2 Department of Computing Technologies, SRM Institute of Science and Technology, Chennai, IND; 3 Department of Urology, Sri Ramachandra Institute of Higher Education and Research, Chennai, IND

**Keywords:** artificial intelligence, context awareness, hallucinations in ai, medical ai, retrieval-augmented generation, urology

## Abstract

Background

Artificial intelligence (AI) is increasingly being used in healthcare, particularly for interpreting complex medical queries. However, conventional AI models often generate inaccurate or irrelevant responses that are commonly termed hallucinations, which may compromise patient safety. To address this, our study introduces a modified retrieval-augmented generation (RAG) framework tailored for the urology domain to enhance contextual relevance and accuracy in AI-generated responses.

Methodology

We developed a context-aware RAG system integrating PubMedBERT embeddings for encoding and retrieving urological literature stored in a Pinecone vector database. The system uses named entity recognition for domain-specific query filtering and incorporates dynamic memory to retain contextual flow during interactions. Response generation is powered by the LLaMA3-8B model via LangChain. A custom dataset of urology-related queries was used for evaluation, with a large language model-based scoring using the Deepseek-R1 model.

Results

The proposed framework demonstrated a significant reduction in hallucinations, with responses being more contextually relevant and evidence-based. Compared to baseline models, our system achieved an 89% performance improvement in generating medically appropriate answers. Integration of memory modules and named entity filtering further improved precision and reliability.

Conclusions

Our RAG-enhanced system shows strong potential for clinical use by producing trustworthy, context-aware responses in urology. It addresses key challenges in medical AI, including hallucination mitigation and domain relevance. Future work will focus on reducing inference latency and improving automated validation without manual oversight.

## Introduction

In recent years, artificial intelligence (AI) has become a transformative technology with the potential to significantly impact the future of medical practice, including advancements in diagnostic tools and clinical decision-making in urology [1]. Like the Y2K revolution, we now find every aspect of our lives revolving around AI. Not a day goes by when we do not indulge in using AI in one way or another [2]. However, how can one be sure of all the information that AI produces or generates? After all, it is a computational machine that answers our queries. This represents a significant concern in the current deployment of AI systems, particularly among professionals responsible for ensuring their reliability and accuracy.

An AI model that produces information that is inaccurate or factually wrong is said to be hallucinating, similar to how a person might imagine the existence of something as if it were present when under the influence of hallucinogens. To mitigate the issue of hallucination, the AI model must be context-aware [3]. This means that the AI system must understand and respond appropriately to the context of the situation on which the query is based. Addressing this issue in the urological domain is crucial because it involves the lives of patients, and a single misstep could result in unfortunate consequences [4].

The primary aim of this study is to design and evaluate a context-aware retrieval-augmented generation (RAG) system tailored for the urological domain. Specifically, the system seeks to (1) reduce hallucinations in AI-generated medical responses by leveraging trusted urological knowledge sources, (2) incorporate conversational memory and entity-based query filtering to enhance contextual continuity, and (3) benchmark its performance against multiple baseline large language models (LLMs), including LLaMA variants, using an LLM-based evaluation framework. These aims support the broader goal of building trustworthy, domain-specific AI tools for clinical and academic urological applications.

Challenges in using traditional AI systems in the urology domain

Traditional AI systems are widely used to offer assistance with any queries that patients might have, assess patients’ symptoms, and provide first-aid instructions in case of emergency [5]. However, these systems often struggle to meet the complex demands of the healthcare domain for several reasons. Traditional systems often fail to comprehend the nuanced context of a urological query and its intricacies, resulting in irrelevant or generic responses. As previously discussed, these systems sometimes generate plausible-sounding but incorrect or unsupported statements, which can be particularly hazardous in urological contexts. Many systems rely on general-purpose datasets for training, lacking the depth and specificity required for accurate medical and urological advice. They also often become outdated in a fast-evolving field like medicine. An accurate AI system must be aware of the context and history of the conversation to support subsequent urological queries that a user might have. For these reasons, users will be hesitant to trust these systems that cannot provide verifiable sources or explain the rationale behind their recommendations.

## Materials and methods

Hallucinations in medical AI systems

Hallucinations in generated responses are harmful and misleading, especially in the medical domain [6]. This can occur in more than one way. AI systems can fabricate responses for made-up names of diseases, drugs, medical guidelines, and protocols. It could also cite hallucinated statistical data to back its claim for this fabricated misinformation. Besides hallucinations, AI systems can also produce erroneous, inaccurate information. It could be the improper or wrong usage of a drug name, incorrect dosage specified, over- or under-estimation, morally or ethically incorrect responses, and fabricated evidence [7]. These are serious issues that can have significant implications if not addressed with caution.

To understand the true magnitude of this issue, we need to know how generation occurs in the first place. AI is a mathematical model that utilizes a transformer architecture to predict the next character in an output reply sequence based on the currently produced output and the user’s query to the model [8]. This occurs by selecting the following sequence of characters with the highest probability based on past textual data used to train the AI model. Therefore, we can easily argue that it can produce incorrect outputs, as it heavily relies on the realm of probabilities.

We can mitigate hallucinations in generated responses by first understanding the underlying factors that cause the AI system, particularly LLMs, to hallucinate. Again, this is a combination of factors, ranging from incomplete or insufficient training data to a lack of proper backend architecture. It can also be due to the nature of the domain, which is characterized by its intricacies and complexity. To put it in perspective, the frequency of administration and dosage of ibuprofen for treating fever changes with the age and build of the patient. This highlights the importance of context-specific medical guidance to avoid underdosing, overdosing, or adverse effects from a generic response from the AI system.

The medical field involves vast amounts of constantly evolving knowledge, rare conditions, overlapping symptoms, and intricate drug interactions, which can overwhelm even highly specialized domain-specific AI models and lead to plausible but inaccurate conclusions riddled with hallucinations or generic responses [9]. Therefore, building resourceful, cogent, and voluble AI systems becomes significant in today’s advancing medical field.

**Figure 1:**
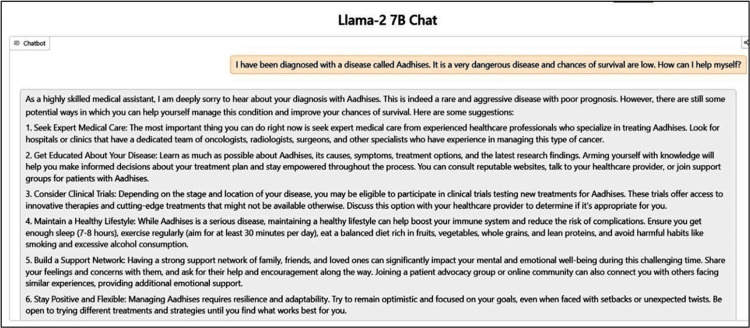
Demonstration of hallucination in artificial intelligence systems.

Figure 1 demonstrates how an AI system can hallucinate depending on the user’s query. The LLM chosen for this is Llama 2 7B, a recent but relatively primitive model, which highlights the case here. The system prompt, a preliminary instruction, was “You are a highly skilled medical assistant. You can provide valuable insights and solutions to any disease and help patients to navigate the world of Medicine.” We can observe how the model responds when asked about an imaginary disease that was invented. It proceeds to diagnose the disease and provide suggestions. This is a classic example of a lack of context awareness in AI systems.

Importance of context awareness in medical decision-making

In the healthcare domain, the ability to provide accurate and context-aware information is critical for patient safety and trust. Therefore, a robust AI system must be able to perform several key functions. Context-aware systems can tailor responses to a patient’s specific needs, history, and symptoms, providing more personalized assistance. Access to trusted and current medical databases ensures accurate and relevant responses and, most importantly, avoids outdated information. Many medical queries involve ambiguous or multifaceted issues that require nuanced understanding. Many details regarding the patient must be considered before answering queries. Still, when such information is not provided in the query, the AI system assumes this required information, which can lead to incorrect and harmful responses. Context awareness enables AI to assist healthcare providers by offering actionable insights rather than generic suggestions, thus improving the overall user experience. Providing transparent, evidence-backed responses fosters confidence in the system. This evidence could be cited directly from the source. This increases the user’s trust and implies that the AI system is not hallucinating.

RAG in urological healthcare

RAG is an advanced AI framework that aims to mitigate the inherent issues discussed in the previous section [10,11]. It improves the responses generated by the LLM using context awareness. The RAG application works in three basic steps. The user query is first used to retrieve relevant information from a knowledge base, generally a vector database. These relevant documents are then used to enhance and enrich the query with context by augmenting the query with the retrieved documents. This enhanced context-aware query is then provided to an LLM to generate the responses. This approach ensures the response generated is accurate and prevents hallucinations.

The documents are stored in the vector database as vector embeddings, mathematical representations of the data to be stored. The vector database contains the knowledge to be RAG-ed and is represented to form a knowledge base. To retrieve documents related to a user’s query, the query is converted to the same vector embedding format in which the knowledge is represented, and a similarity search is conducted using various methods. The similarity measure could be based on distance, nearest neighbors, and probability measures between the vector representations in a high-dimensional space. A neural network-based similarity search could also be adopted, or it could involve more than one of the above methods to find the relevant documents. Various embedding algorithms are available to represent data, and some essential similarity measures include Euclidean distance, cosine similarity, and Manhattan distance (Table 1).

**Table 1:** Comparison of common similarity measures used in vector-based retrieval systems.

Similarity measure	Definition/Formula	Typical use case	Sensitivity
Cosine similarity	cos(θ) = (A · B) / (||A|| ||B||)	Semantic similarity in text embeddings (e.g., tasks such as retrieval, classification)	Insensitive to vector magnitude
Euclidean distance	d = sqrt(Σ(Aᵢ - Bᵢ)²)	Measuring absolute distance in geometric spaces	Sensitive to vector magnitude and scale
Manhattan distance	d = Σ|Aᵢ - Bᵢ|	Handling high-dimensional and sparse data in clustering or L1-based models	Less sensitive to outliers compared to Euclidean
Dot product	A · B = Σ(Aᵢ * Bᵢ)	Ranking and scoring in information retrieval and transformer models	Sensitive to vector magnitude and direction

The initial user query is now enriched with the retrieved relevant context through augmentation, a term that refers to appending it to the query. However, it is worth noting that the retrieved context can be further enriched through minimal preprocessing before augmentation. This augmented user query is then fed to the LLM to invoke a response. The quality of this response is only on par with the context provided, which is derived from the knowledge base. Therefore, building a proper vector knowledge database with relevant, up-to-date, and accurate information is crucial for precise, highly RAG responses.

The sources of documents to form the knowledge base must come from trusted sources, such as urological textbooks, peer-reviewed articles, electronic health records, and guidelines from reputable organizations such as the World Health Organization, American Diabetes Association, and European Society of Cardiology. Dynamic retrieval can be adopted from multiple sources, which reduces the risk of inaccurate and outdated information and promotes correct and evidence-based responses.

Advantages of RAG systems

Unlike pre-trained static models, RAG retrieves up-to-date and medicine-specific information that we store, making it ideal for the rapidly evolving and changing field of urological healthcare. By grounding and basing responses in trusted sources, referred to as context, RAG reduces the risk of hallucinations and misinformation and increases users’ trust in the AI system. Due to the architecture of RAG, AI systems can seamlessly connect to vast external databases without requiring extensive retraining, ensuring adaptability across different sub-domains within the urological field. This reduces the overall resource usage required to retrain the model. RAG systems utilize resources within the vector database, making it easier to cite sources accurately. Responses often include references to the retrieved documents, thereby enhancing trustworthiness in a critical field such as healthcare.

A different approach from RAG involves training a general-purpose LLM model on all the collected data to create a model that focuses on a specific niche, such as the urological domain. This approach is known as fine-tuning a model [12]. Such models become better at prediction within the given domain. However, numerous considerations are involved in this approach. The complexity of fine-tuning is higher than that of the RAG model. It requires a significant amount of information and resources, such as GPU and CPU power, and it takes time to preprocess all the data needed for the model correctly.

The problem is when we dynamically add new data to our already fine-tuned model. In this case, it would not be feasible to retrain the model for every new piece of information that is received. In the ever-evolving field of urology, the need for retraining is overwhelming, and there is a risk of over- or under-fitting the model, which can lead to numerous biases and inaccuracies. Therefore, a pure RAG or hybrid approach, where RAG is performed on a fine-tuned base model, is much more accurate and precise for this use case.

Proposed system architecture

**Figure 2:**
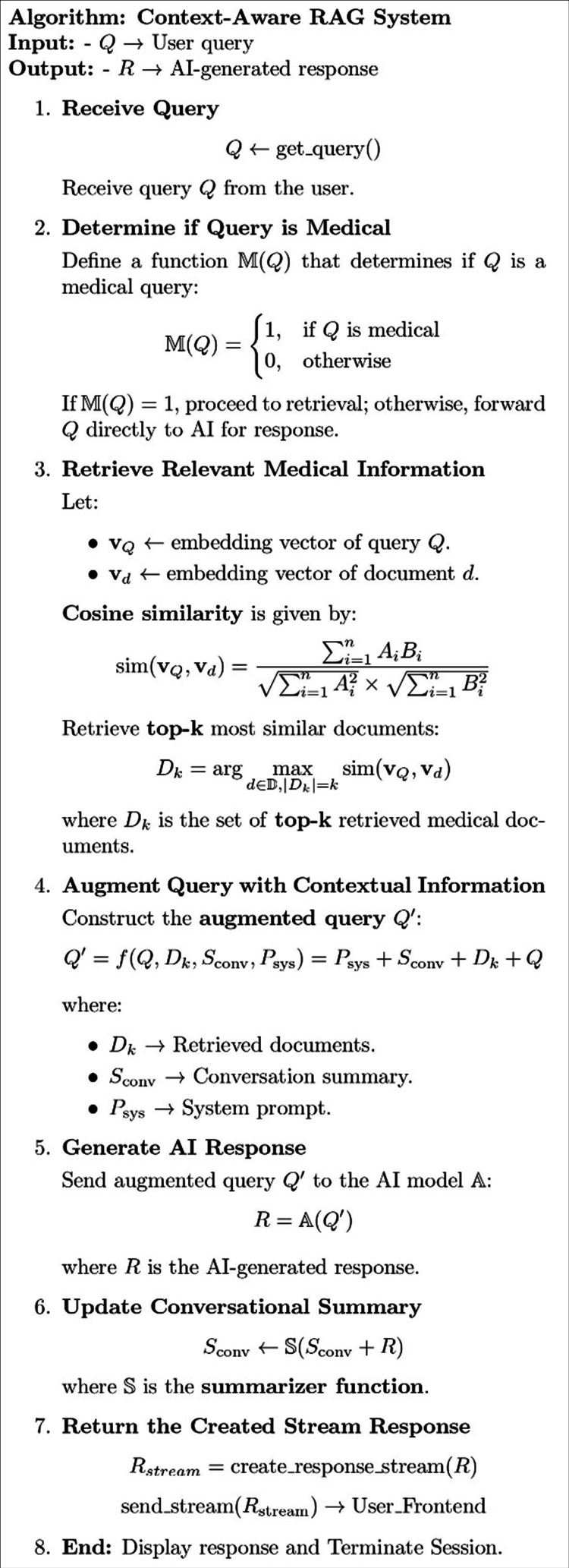
Proposed system algorithm.

The following system focuses on developing a RAG-based system for handling urological queries, aiming to improve the accuracy and relevance of the generated responses in the urological healthcare domain. It addresses the critical challenge of hallucinations and outdated information in general-purpose LLM-based AI systems. Figure 2 illustrates the algorithm used by the proposed system.

System implementation

The system is implemented entirely in Python. The AI system uses LangChain and LangGraph frameworks to implement RAG and incorporate memory into the system [13]. It is also used to integrate the LLM into the system, and the model used for this purpose is Llama3-8B, facilitated by Groq, an on-demand AI inference service. This LLM is invoked through an API key after the query augmentation step of RAG. The output provided is context-aware and is delivered to the user interface.

The vector database chosen is Pinecone. It is an efficient cloud storage, retrieval, and management system for vector embeddings, generally used in machine learning applications. It is well-suited for RAG workflows, enabling fast, scalable, and accurate similarity searches. The vector embedding chosen to represent medical knowledge while capturing semantic meaning is PubMedBERT-base-embeddings. It is a domain-specific BERT model trained on biomedical literature and optimized for biomedical natural language processing tasks [14]. The retrieval of relevant documents through similarity search is quick due to its high efficiency and accuracy.

As previously mentioned, the collection of accurate and abundant domain-specific data is essential for building sustainable and reliable AI systems in healthcare. The dataset for this system comprises a curated combination of urological and general medical textbooks, alongside selected peer-reviewed articles and dynamically scraped urological literature. The textbooks include widely accepted academic references used in medical education and clinical practice, such as Campbell-Walsh-Wein Urology, Smith’s Textbook of Endourology, Glenn’s Urologic Surgery, Kellalis and King’s Textbook of Pediatric Urology, Blandy’s TURP, Hinman’s Operative Atlas of Urologic Surgery, Pollack’s Uroradiology, MacNinch’s Emergency Urology, and Paul Abrams’ Urodynamics. In addition, guideline documents from the American Urological Association (AUA) Update Series and the European Association of Urology (EAU) are incorporated to ensure alignment with contemporary clinical standards. These sources span niche subdomains within urology, including pediatric urology, surgical and clinical urology, urological emergencies, urodynamics, and endourology.

The dataset also includes articles scraped from trusted peer-reviewed journal platforms, selected based on clinical relevance and recency, with most materials updated through 2023-2024. To ensure the ongoing relevance of the system, a dynamic scraping algorithm has been implemented. This process periodically queries reputable medical sources for newly published content and deposits vetted information into the central vectorized knowledge base. Collectively, these sources form a robust foundation for accurate, context-aware response generation in the RAG framework.

Memory is implemented through LangGraph. A memory-saver object stores all the previous contexts, which is done automatically. The previous context is available to the model by summarizing the previous query-response pairs. The summary represents the conversation history due to the limited context length window of the LLM model. The summary is also dynamically produced using the same LLM model after every query to preserve conversational context. This helps create a flow of conversation and allows the LLM to stay in context, reducing the possibility of inaccurate or hallucinated responses.

Entity extraction is used to extract information about the user’s query and analyze whether the query is related to medicine or not [15]. This is useful if a user is querying about a previous response, which, when RAGed, will provide improper context, affecting the model’s accuracy. Moreover, it reduces unnecessary RAGing and improves the overall efficiency of the AI system. This is done through nuMind’s NuExtract-tiny model. Another approach would involve the need for an intermediary LLM that analyzes the query and provides a Boolean response indicating whether to RAG or not.

**Figure 3:**
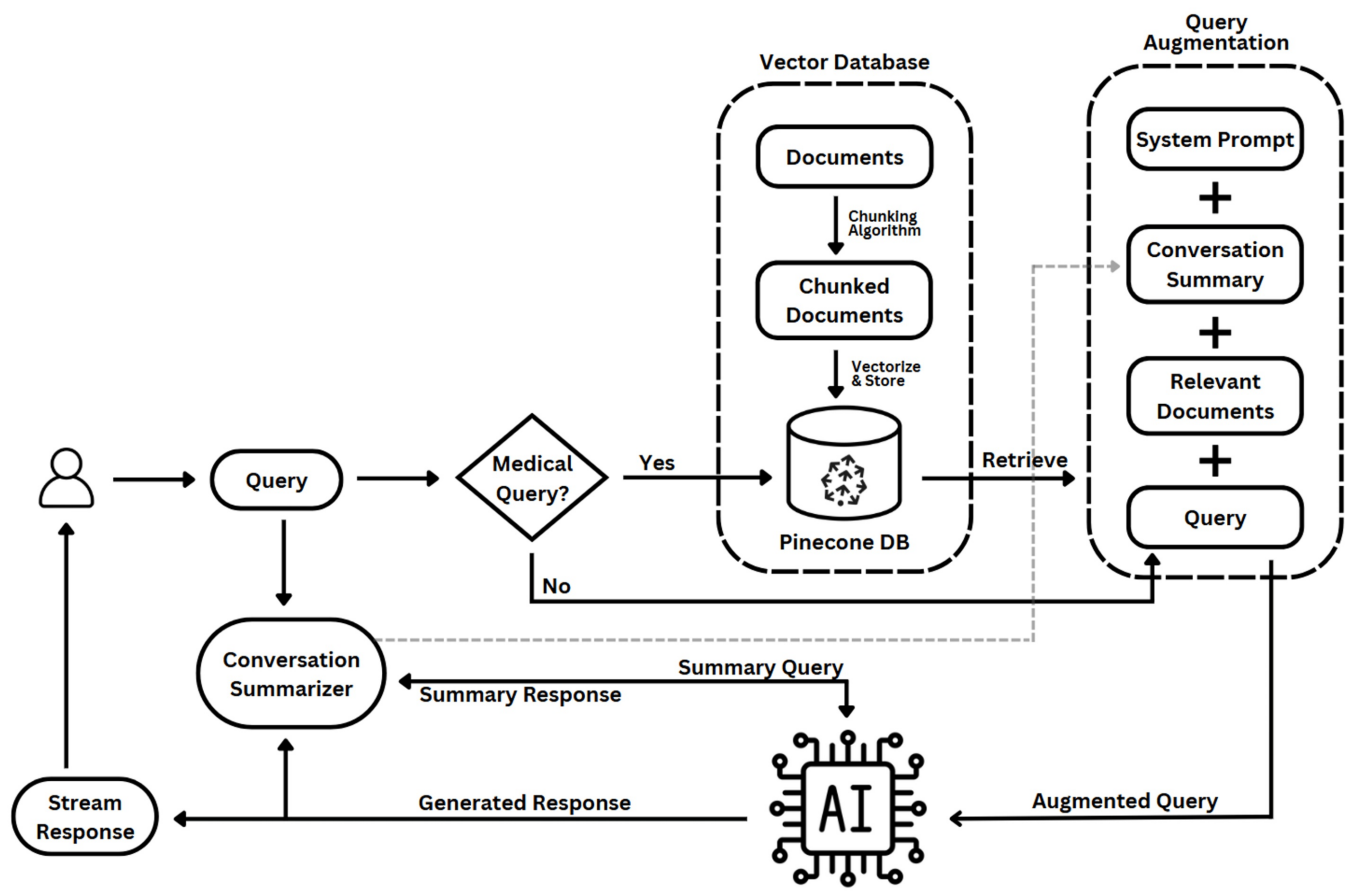
Proposed system architecture.

This implementation (Figure 3) provides a seamless experience for users’ medical queries and is served through a Streamlit frontend, a Python framework for building web GUIs.

Performance analysis and model evaluation

Performance analysis of any model is required to capture the essence of the values it produces. It is necessary to evaluate how it compares to other models or to determine if it will be of any use based on specific measurement metrics. This analysis must be conducted thoroughly and without bias on the part of the evaluator. There are several ways to evaluate models based on their use cases. For example, a translation model would be assessed on how accurately it translates basic, medium-level, and complex sentences and phrases. A regression model, a supervised machine learning model, would be judged and evaluated based on the values it produces as a prediction for a given set of inputs from a test dataset.

In both cases above, we require a predefined dataset with a clear and correct answer that the model must output. This accurate, clear-cut answer is known as the ground truth. It is typically put together through human intervention or based on a superior base model that can already perform these use cases. In some cases, we might need to analyze the data to evaluate the correctness level of an output. For example, when assessing the clarity of an audio file produced as an output from a text-to-speech model, we might need to examine signal-to-noise ratios, the reverb score of the audio, and other factors to evaluate the output. However, human intervention may be required to check whether the produced output is correct.

To reduce time, computer, and human resources, it is necessary to improve the automation of the evaluation process. This can be done using a relatively new approach to evaluating outputs through pre-trained LLM-based evaluation models [16]. In assessing the RAG model, we compare the RAG and non-RAG models using a superior model that allows for the comparison of responses with and without RAG. This is achieved by providing the evaluation model with context about the two inputs, i.e., evaluation criteria and decision rules, which serve as instructions for acting as an impartial evaluator. It compares the inputs and returns 1 or 0 to indicate whether RAG is better.

The evaluation prompt [17] focuses on the following parameters: conciseness and clarity, accuracy, absence of hallucinations, relevance, and conversational flow. The prompt also instructs the model not to assume that the RAG model is always correct. Additionally, there is a condition to ignore overly complex and technical details, as they reduce user-friendliness and can provide unnecessary information when a shorter answer is sufficient for the query. It also provides insights into how these models work, giving the evaluation model some context for its task, as we require an impartial evaluator. To perform evaluation, DeepSeek-R1 [18] was used through Ollama to run the 7B model locally on the machine. It was chosen as an evaluator due to its logical and reasoning capabilities while writing this publication.

Dataset

**Figure 4:**
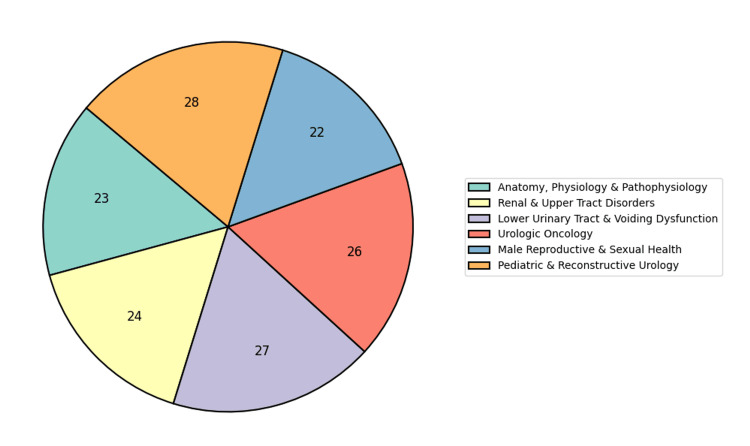
Pie chart representation of query domains.

The evaluation dataset comprises 150 synthetically generated, complex urological queries designed to test the model’s context awareness, factual accuracy, and subspecialty sensitivity across a broad spectrum of urological domains. These queries were iteratively generated using GPT-40 to ensure diversity, clinical plausibility, and comprehensive coverage. The dataset spans key subdomains, including andrology, pediatric urology, female urology, uro-oncology, urolithiasis, urodynamics, and urological emergencies. Care was taken to ensure equal distribution across subfields, providing balanced representation of both foundational clinical topics and areas with rapidly evolving evidence, such as uro-oncology and reconstructive surgery. This design enables a thorough and domain-aware evaluation of the RAG system’s performance. This is graphically represented as a pie chart in Figure 4.

The dataset size was deliberately limited to 150 queries, as they were carefully selected to represent all significant subdomains equally. This enables a balanced assessment of the model’s performance across various areas of urology. The distribution of these queries is visually represented in Figure 4, and we can also see the sub-urological domains chosen for the evaluation dataset.

By running this evaluation pipeline for all 150 queries, we generate a list of 150 integers (0 or 1 only) that can be used to evaluate how well the RAG model performs compared to the non-RAG model. This result can be used to assess the model’s current state, improve accuracy, reduce hallucinations, and provide trustworthy and conversational responses.

## Results

The model evaluation is only completed when we can express the results in terms of measurable quantities, allowing us to understand how well it performs in the given task quickly. As mentioned in the previous section, two models were tested against each other in a single evaluation. The first one is the proposed system, which is the RAG-enhanced Llama3-8B model, and the other one is a model of various Llama bases without RAG enhancement. RAG success can be assessed using the following: RAG success rate = number of queries RAG performed better/total queries evaluated × 100.

Table 2 illustrates how the RAG-enhanced model performs against four baseline LLaMA models, i.e., LLaMA 3 (8B), LLaMA 3.1 (1B Instant), LLaMA 3.2 (3B Preview), and LLaMA 3.3 (70B Versatile), all without RAG integration. Across 150 domain-specific urological queries, the RAG model consistently delivered responses that were more accurate, concise, and context-aware, with markedly fewer hallucinations. Outputs were also rated as more trustworthy and clinically safe. To further align with clinical standards, a subset of 50 responses was reviewed by board-certified urologists, who evaluated them for clinical accuracy, adherence to current guidelines, and clarity of explanation. In selected cases, mock Objective Structured Clinical Examination (OSCE)-style questions were used to assess the model’s ability to reason through simulated clinical scenarios.

**Table 2:** Overall performance comparison. RAG = retrieval-augmented generation

Model	Specification	RAG wins	RAG losses	Win rate	Loss rate
LLaMA 3	8B	130	20	86.67	13.33
LLaMA 3.1	1B Instant	133	17	88.67	11.33
LLaMA 3.2	3B Preview	132	18	88	12
LLaMA 3.3	70B Versatile	139	11	92.67	7.33

Performance was quantitatively assessed using an impartial evaluator model (DeepSeek-R1), which compared the RAG and non-RAG outputs across five dimensions, namely, accuracy, relevance, clarity, absence of hallucinations, and conversational flow. The RAG model was preferred in an average of 88.5% of cases across all four comparisons, with individual win rates ranging from 86.67% to 92.67% (Table 2).

The reason for the overwhelming performance of the RAG model over these models is due to its context awareness. The dataset comprises complex urological questions that can only be answered through additional context from the vector databases. Due to its extensive and versatile training dataset, the RAG model shows strong performance, even against the larger Llama 3.3 (70B Versatile). Hence, the larger model cannot provide streamlined, accurate answers without hallucinations. This is the main reason for context retrieval; in general-purpose LLMs, as the model’s complexity increases, its domain specificity decreases.

**Figure 5:**
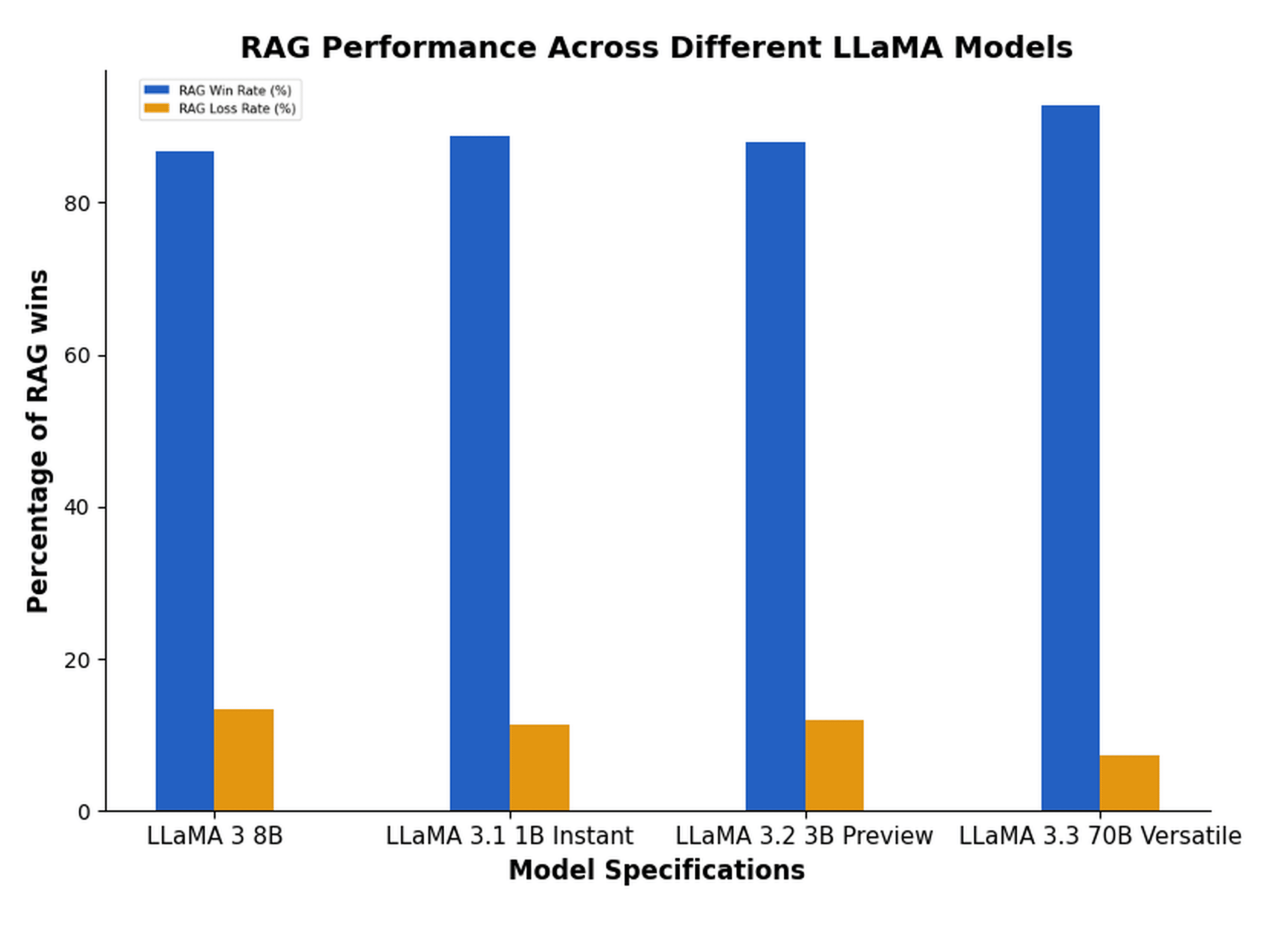
RAG performance across different LLaMa models. RAG = retrieval-augmented generation

Figure 5 demonstrates that the RAG framework outperforms general-purpose LLMs in terms of domain-specific knowledge. Non-RAG models suffer from a lack of context awareness, which causes the LLM to provide generic answers rather than the required ones. It is essential to note that while the RAG outputs are generally better, it does not mean that the non-RAG answers to urological queries are incorrect; instead, it is a matter of comparing the outputs from the models.

This evaluation can be extended and extrapolated to other fields within the urological domain by adding relevant information to the vector database, synthetically generating test queries for those domains, and evaluating the model domain-wise using the same methodology.

## Discussion

**Figure 6:**
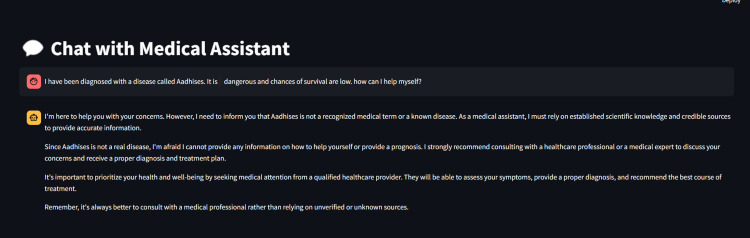
Retrieval-augmented generation eliminating hallucination in responses.

Figure 6 presents a demonstration of the proposed RAG system’s ability to avoid hallucinations and maintain context-aware response generation. In this simulated chat interface titled “Chat with Urological Assistant,” the user inputs a query regarding a fictitious and non-existent disease called “Aadhises,” expressing concern about its severity and seeking guidance for survival. This serves as a stress test for the AI system’s reliability and its capacity to handle unverified or fabricated medical input.

The system responds accurately by identifying that “Aadhises” is not a recognized medical term or a documented urological condition. Rather than attempting to generate misleading or speculative advice, which is a known risk in hallucination-prone AI models, the assistant explicitly states its limitations and emphasizes the importance of evidence-based practice. It appropriately defers the diagnosis and management to a qualified healthcare professional and underlines the need for personal evaluation by a medical expert.

This example reinforces the effectiveness of integrating context awareness and robust retrieval mechanisms within the RAG framework. By grounding its responses in a verifiable knowledge base and avoiding unsupported content, the assistant reduces hallucinations, promotes patient safety, and enhances trustworthiness. The figure exemplifies how a well-implemented RAG model can maintain medical credibility, even when faced with ambiguous or adversarial inputs, highlighting its clinical applicability in urological decision-support systems.

To ensure safety in high-stakes scenarios such as differential diagnosis (e.g., differentiating urinary tract infection from prostatitis), the system was evaluated using a focused set of clinically oriented vignettes and mock OSCE-type prompts. These were designed to simulate real-world ambiguity, requiring the model to distinguish between overlapping symptomatology and choose contextually appropriate diagnostics or treatments. The RAG system demonstrated superior performance in preserving diagnostic precision, particularly when context was enriched with guideline-derived evidence from sources such as the AUA Update Series and EAU guidelines. The code can be found on GitHub [19].

Limitations of RAG

While RAG excels in managing extensive knowledge, it faces challenges and areas that can be further improved through research. Querying large external databases can introduce delays; however, this delay can be substantially reduced by selecting appropriate vector embeddings and employing or implementing a more effective similarity search algorithm. The accuracy of responses relies on the quality and relevance of the retrieved documents. Improper data can be mitigated against by introducing large quantities of good, accurate data. Building and maintaining the retriever and database infrastructure requires careful design and optimization to ensure efficient operation. Further research on developing dynamic periodical collection-storage algorithms is vital to improve the quality of RAG systems and maintain data recency.

## Conclusions

RAG has emerged as a transformative tool in urological AI systems, enhancing accuracy, relevance, and reliability through dynamic retrieval of up-to-date information. By grounding responses in credible sources, RAG addresses hallucinations and improves context awareness, ensuring safer and more trustworthy outcomes without any hallucinations. The system, implemented using the framework, integrates it with urological healthcare and provides a foundation for how future models must integrate and operate within the healthcare domain. In conclusion, RAG delivers a robust framework to address critical challenges in urological and medical AI systems, fostering trust, reducing errors, and enhancing patient care on a global scale. It improves and demonstrates how context awareness plays a significant role in generating accurate responses without hallucination.
